# MiRNAs Are Involved in Tall Cell Morphology in Papillary Thyroid Carcinoma

**DOI:** 10.3390/cancers11060885

**Published:** 2019-06-25

**Authors:** Laura A. Boos, Anja Schmitt, Holger Moch, Paul Komminoth, Cedric Simillion, Ilaria Marinoni, Yuri E. Nikiforov, Marina N. Nikiforova, Aurel Perren, Matthias S. Dettmer

**Affiliations:** 1 Institute of Pathology, University of Bern, Murtenstrasse 31, CH 3008 Bern, Switzerland; laurie.boos@googlemail.com (L.A.B.); anja.schmitt@pathology.unibe.ch (A.S.); ilaria.marinoni@pathology.unibe.ch (I.M.); aurel.perren@pathology.unibe.ch (A.P.); 2 Department of Pathology and Molecular Pathology, University Hospital Zurich, Rämistrasse 100, 8091 Zurich, Switzerland; holger.moch@usz.ch; 3 Institute of Surgical Pathology, Stadtspital Triemli, Birmensdorferstr. 497, 8063 Zürich, Switzerland; Paul.Komminoth@triemli.zuerich.ch; 4 Department of BioMedical Research, University of Bern, Murtenstrasse 31, CH 3008 Bern, Switzerland; cedric.simillion@protonmail.ch; 5 Department of Pathology and Laboratory Medicine, University of Pittsburgh Medical Center, Pittsburgh, PA 15213, USA; nikiforovye@upmc.edu (Y.E.N.); nikiforovamn@upmc.edu (M.N.N.)

**Keywords:** thyroid carcinoma, miRNA, PTEN, VEGF, prognosis, tall cell, TERT

## Abstract

Five percent of papillary thyroid carcinomas (PTC) show an adverse clinical outcome (ACO). The tall cell variant of papillary thyroid carcinomas (TCV) is a good predictor of an ACO, however, the identification of tall-cells is subjective. Micro RNAs are short non-coding ribonucleic acids (miRNA). Their expression in PTC could be a powerful, more objective predictor of prognosis. Methods: Forty-four PTC underwent miRNA profiling, twenty-four of them were TCV. The miRNA dataset was validated by analysis of expression of known target proteins (vascular endothelial growth factor (VEGF) and phosphatase and tensin homolog (PTEN)) in 125 patients including 48 TCV and 57 with an ACO. Results: One hundred and forty-nine miRNAs were significantly associated with an ACO, seventy-one of them with TC-morphology. Twenty-two miRNAs were identified as targets for VEGF and thirty-two as targets for PTEN. In univariate and multivariable analysis, reduced expression of PTEN and an increased expression of VEGF were associated with shorter relapse free survival. A classifier, including TC-morphology, pT-stage, VEGF, and PTEN, predicted relapse with an 80% accuracy. Conclusions: Some miRNAs predict outcome in PTC and are involved in TC-morphology in PTC. These miRNAs may serve as more objective indicators of an ACO than tall cell morphology. PTEN and VEGF protein expression are prognostically relevant and are at least partially regulated by miRNAs.

## 1. Introduction

Thyroid carcinoma accounts for about 1% of all human malignant neoplasms [1]. Eighty-six percent of these carcinomas are papillary thyroid carcinomas (PTC). These belong to the group of differentiated thyroid cancers, which is known for its favorable prognosis [2] in contrast to poorly differentiated or anaplastic thyroid carcinomas [3,4,5]. The standard therapy for PTC is thyroidectomy followed by a radioiodine therapy (RAI), which generally cures the patient [6]. Nevertheless, 5% of PTC encountered by clinicians and pathologists do not follow this favorable course but rather behave more aggressively [7]. Currently, there are several features known to correlate with such an adverse outcome like pT-stage (gross extrathyroidal extension and tumor size) and patient age [2]. Furthermore, morphological subtypes such as tall-cell variant have been identified as adverse prognosticators [7,8]. Although these features are recognized for patient management, the prediction of the clinical behavior in an individual case remains difficult.

We have recently shown that even a small percentage of tall-cell (TC) morphology is of strong prognostic importance [7]. Molecular markers to reliably identify TC are not known and the biology behind this morphological phenomenon remains mostly elusive. A deeper understanding of the disease would be important, since the identification of TC versus non-TC in a given tumor can be subjective and is hampered by lack of experience and inter-observer variability.

Telomerase reverse transcriptase (TERT) promoter mutations are known to be a strong predictor of an adverse outcome. However, the mutation is only found in about 7–8% of PTC of which all are TC variants in our previous study [7]. Additional objective tools to reliably predict an adverse clinical outcome (ACO), independent of TC morphology would be helpful.

Micro RNAs are short non-coding ribonucleic acids (miRNA). They are a class of non-coding 18–24 nucleotide long RNAs that play an important epigenetic regulatory role in nearly all cellular processes including cancer development. They can either function as oncogenes, so called oncomiRs or as tumor suppressing miRNAs by negatively regulating the gene expression of their target genes [9]. Their expression is tissue specific and so, specific tumor signatures have been developed for various human malignancies including thyroid cancer and its subtypes [10,11,12,13].

Phosphatase and tensin homolog (PTEN) is known to play an important role as a tumor suppressor gene in human cancers and thyroid carcinomas exhibit a decreased expression of PTEN [14]. Amongst other functions, PTEN inactivates the Phosphoinositide-3-kinase/Protein kinase B pathway, leading to tumor progression via the Phosphoinosito 3-kinase (PI3K)/Protein Kinase B(AKT) pathway [15].

Vascular endothelial growth factor (VEGF) is a known stimulator of endothelial cell proliferation and migration [16]. While some studies have suggested an aggressive clinical course in PTC with VEGF overexpression, this could not be confirmed by others [17]. New VEGF-A and multi-tyrosine kinase inhibitors like Lenvatinib are now on their way to the clinics and thus are a promising tool in RAI refractory tumors [18].

In this study, we performed a comprehensive miRNA profiling to identify transcriptomic changes on miRNA level in PTC and to evaluate if some of them could add prognostic value in individualized patient management, independent from TC morphology. Further, we analyzed whether those miRNAs may be involved in the development of TC morphology. We then used protein data of PTEN and VEGF, two genes which are frequent targets of the found miRNAs to cross validate our miRNA expression data. Lastly, we explored the prognostic role of these two genes in PTC.

## 2. Results

### 2.1. MiRNA Analysis

In total, 149 miRNAs were statistically significantly associated with a decreased relapse free survival (Table 1).

MiRTarBase plus literature research was used to identify experimentally verified miRNA targets of VEGF and PTEN [19]. An amount of 4076 miRNAs are currently listed in miRTarBase as verified target-mRNA interactions of different genes and 125 miRNAs are listed as targets for PTEN and 122 for VEGF.

The bioinformatic target prediction tool mirDIP was used to identify miRNAs targeting PTEN and VEGF [20]. When a miRNA was predicted in at least two different miRNA databases by mirDIP and it was deregulated in the correct direction (upregulated), the Kaplan–Meier test for a decreased relapse free survival in our dataset was run. This bioinformatic pipeline identified 22 miRNAs in our dataset targeting VEGF and predicting a decreased relapse free survival (RFS; Table 2). Fifty four percent of these VEGF-targeting miRNAs are also listed on the miRTarBase website. The same approach for PTEN identified 32 miRNAs (Table 3) in total and 28% of them are listed in miRTarBase and in our dataset. Applying the chi-square test to these numbers reveals that the miRNAs that were identified by us are significantly overrepresented in miRTarBase (*p* <0.01 for VEGF and PTEN).

Seventy-one out of 149 miRNAs associated with a decreased RFS were significantly correlated with TC. Among them are many well-known cancer-associated miRNAs like miR-1, miR-21 or miR-146b-5p (Table 1).

The Kruskal–Wallis Test with Bonferroni correction identified nine of our 754 miRNAs tested to correlate significantly with our VEGF expression and for PTEN, we could find 11 miRNAs with this approach (Table 4) which is a highly significant overrepresentation of miRNAs identified (*p* <0.01). In addition, all those miRNAs are predicted as VEGF, respectively PTEN targets in multiple (3 to 18) different miRNA databases according to miRDIP [20].

Survival- and miRNA data were extracted from the TCGA dataset [21]. After correction for multiple testing, no single miRNA was found to be significantly associated with a decreased relapse free survival. This is reflected by a low statistical power with only 17 cases of PTC with an ACO in the TCGA.

### 2.2. Immunohistochemical Analysis

PTEN protein expression was preserved in all patients of the control group (CG), whereas it was lost in >50% of the ACO patients. VEGF on the other hand was preserved in about 40% of the control cases while it was only expressed in 2% of the ACO patients. The detailed immunohistochemical results are shown in Table 5.

**Figure 1:**
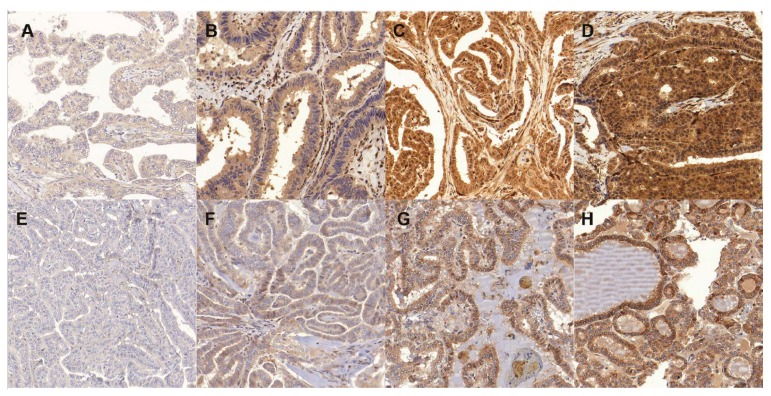
Immunohistochemical expression of PTEN (**A** negative, **B** weak; **C** moderate, **D** strong) and VEGF (**E** negative, **F** weak; **G** moderate, **H** strong) in papillary thyroid carcinoma (PTC).

**Figure 2:**
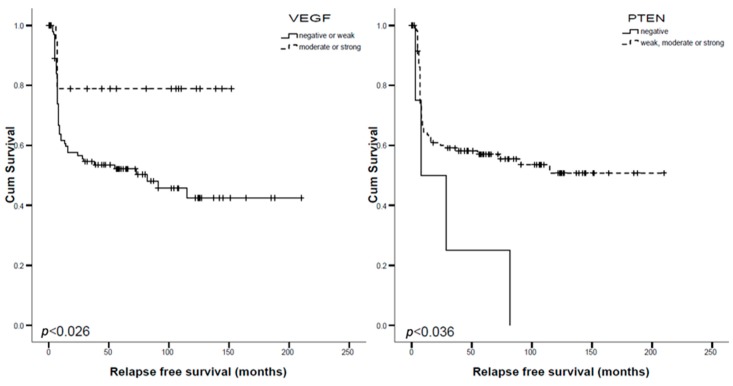
Kaplan–Meier Analysis for VEGF (**left**) and PTEN (**right**) and relapse free survival.

PTEN and VEGF-A were able to predict patient relapse on univariate analysis (Table 5, Figure 1 and Figure 2). Multivariate analyses, including tumor stage, age, gender, and TC morphology as covariables were run and confirmed their independent prognostic role: PTEN (*p* = 0.002; Exp(B) 0.176) and VEGF (*p* = 0.031; Exp(B) 3.118).

Further, VEGF protein expression was significantly positively associated with increased patient age (*p* <0.014).

### 2.3. TERT Promoter Mutation Analysis

As reported [7], we analyzed 53 tumor samples in the ACO group of which 8 had a TERT promoter mutation, while 57 samples of the control group were TERT promoter mutation negative (*p* < 0.002).

TERT promoter mutational status correlated neither with VEGF nor with PTEN protein expression in univariate analysis, the factors which turned out to be independent from TC morphology. Running TERT in a multivariate analysis with PTEN including tumor stage, age and gender as covariables, TERT (*p* = 0.002; Exp(B) 3.737) and PTEN (*p* = 0.036; Exp(B) 0.259) were independent parameters. On repeating the analysis, replacing PTEN with VEGF, both parameters were significant as well: TERT (*p* = 0.019; Exp(B) 2.524) and VEGF (*p* = 0.038; Exp(B) 0.288).

### 2.4. Histopathological Evaluation

All cases were carefully re-examined by three independent pathologists (MSD, AS, AP). In cases of discrepancy, a consensus diagnosis was rendered at a multiheaded microscope. The amount of TCs was semiquantitatively assessed as previously described [7]. The ACO group consisted of 38 classic PTC, 15 PTC of follicular variant, and 4 PTC of a special variant of which 36 had at least 10% of TC as published [7]. Sixty-eight PTC cases without relapse, composed of 30 classic PTC, 32 FVPTC and 6 PTC of a special variant served as control group including 12 cases with at least 10% TC (Table 6). Twenty-one tumors had lymph-node metastases and 6 had distant metastases.

### 2.5. Predictive Algorithm

**Figure 3:**
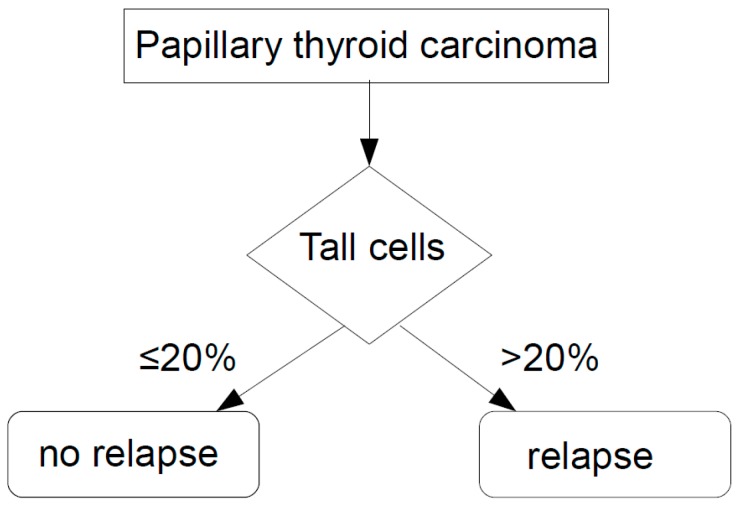
decision tree with tall cells (TC) to determine relapse free survival (RFS) in PTC, overall accuracy 73.6%.

By feeding TC, TERT promoter mutation status, patient age, and gender into the SPSS decision tree CRT analysis tool, an algorithm was developed to render a diagnostic accuracy to predict patient relapse of 73.6% only using TC-Morphology (Figure 3).

**Figure 4:**
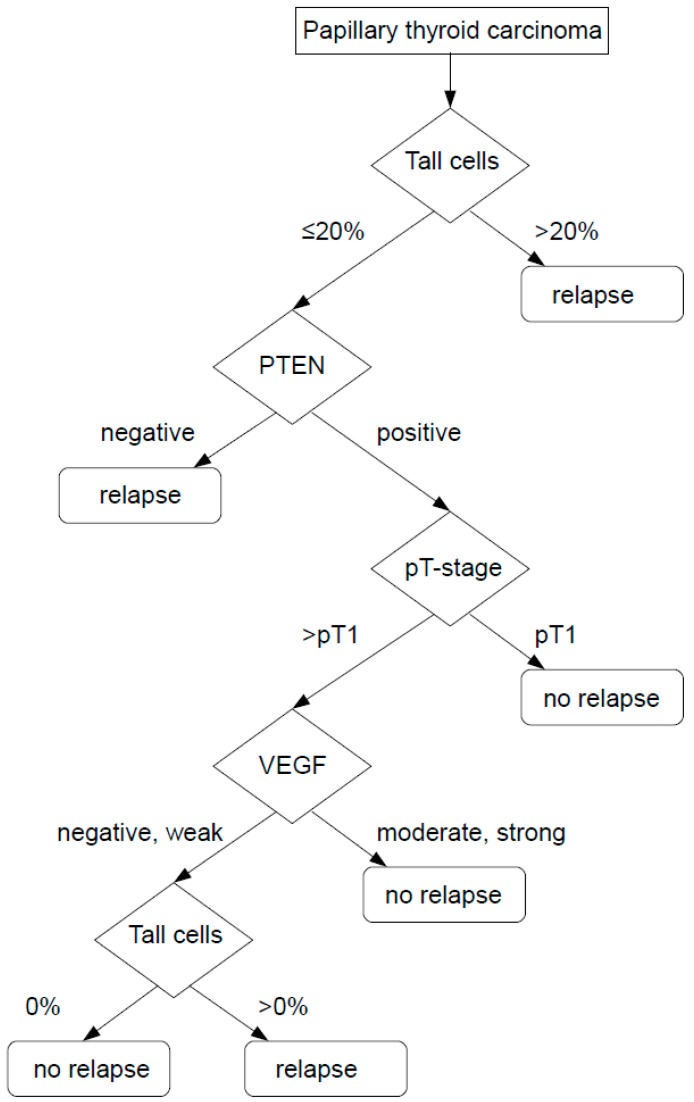
decision tree with TC, PTEN, VEGF and pT-stage to determine RFS in PTC, overall accuracy 79.2%.

Adding the two immunohistochemical markers PTEN and VEGF into the calculations improves the overall accuracy to 79.2% and the accuracy of tumor relapse prediction increases almost 15% up to 67% (Figure 4).

## 3. Discussion

In the present study we searched for epigenetic changes on the miRNA level that were able to predict patient outcome and identified a subset of those miRNAs to be potentially involved in TC phenotype of PTC.

### 3.1. MiRNAs and Outcome

MiRNAs are small non-coding RNAs that regulate about two thirds of the human genome. They are centrally involved in epigenetic gene regulation in basically every biological process as well as in carcinogenesis, including thyroid cancer [11,12,22]. After extensive miRNA profiling, we provided the first comprehensive list of in total 149 miRNAs associated with a decreased RFS in PTC.

The identification of miRNAs, which indicate an adverse outcome is not only interesting from a scientific perspective but also from a diagnostic and prognostic point-of-view. This could help in diagnostically difficult cases in which tall cells as a well-established morphological tool to predict an adverse outcome are not identified [7] and bring a more objective tool into the hand of a diagnostic pathologist than the subjective identification of a small subpopulation of TC.

### 3.2. Validation of Dataset

Some of the miRNAs like miR‑1, miR-23b, miR‑34b, miR‑146b, or miR-150 have already previously been identified as markers of aggressiveness in thyroid cancer and could be confirmed in the present study, underscoring the validity of our analysis [13,23]. Nevertheless, we aimed for a further, more comprehensive validation of the miRNA data.

For this, we looked into genes which are known to play an important role in thyroid pathogenesis and could be potentially regulated by those miRNA changes, and identified PTEN and VEGF. In fact, a large number of miRNAs which were identified by us are predicted by various miRNA target prediction databases to have PTEN or VEGF as a target and could be experimentally verified in thyroid cancer and various other tumor types [20,24]. The miRNAs identified are significantly overrepresented in the miRTarBase database of experimentally verified miRNA-protein targets [24]. If our miRNA data had not been in an appropriate biological context, we would not have been able to find so many experimentally verified miRNAs among the miRNAs which we identified to target VEGF and PTEN based on our profiling data.

In addition, we found a significant correlation between PTEN and VEGF expression and deregulated miRNAs in our dataset with a significant overrepresentation of known miRNA–VEGF/PTEN interactions. While a thorough experimental validation is beyond the scope of this work, we believe that this lays a solid foundation for an informed approach for further studies in the field.

Of note, we also aimed to validate our miRNA profiling data results with the largest miRNA PTC dataset available, the TCGA dataset [21]. The bioinformatic analysis revealed that not a single significant miRNA predicted a decreased RFS in the TCGA dataset. We believe the reason is that the statistical power in the TCGA concerning patients with an ACO is too low. To our knowledge, there is no other dataset available for a direct cross-validation.

### 3.3. PTC-TC

PTC is the most common type of thyroid carcinoma. It is known to have a favorable prognosis and is generally cured by partial or total thyroidectomy following RAI [6]. Nevertheless, a small number of patients suffers from relapses and would therefore profit from a closer follow-up care. Although several features have been discovered that correlate with a more aggressive clinical behavior, not all patients that will show a relapse can be identified [7,25]. In a previous work, we showed that only 10% of TCs in a single tumor are one of these strong predicting factors [7].

The identification of TC morphology seems easy at first hand but in daily routine it is nevertheless often missed. This holds especially true when the percentage of TCs is low. Obviously, there is some subjectivity in judging TC morphology. Therefore, the identification of biomarkers that help to gather reliable prognostic information and where the read-out is more objective is of clinical importance.

### 3.4. PTEN

Altered PTEN expression plays an important role in human cancer development. Mutations of PTEN have been detected in various other types of human carcinoma including breast cancer, endometrial and thyroid carcinoma [14,26,27]. Despite this well-established knowledge, the prognostic role of PTEN has to our knowledge not yet been systematically evaluated in a large cohort of PTC patients. As expected, we found a decreased relapse-free survival in the case of PTEN protein loss. Interestingly, this proved to be an adverse prognostic marker independent of TC morphology in a multivariate analysis.

We found a loss of PTEN protein expression in more than 50% of cases in the ACO group. Since only 2–5% of PTC harbors a PTEN mutation according to the TCGA dataset and other studies [2,21], its loss of protein expression cannot be explained by mutations alone. One reason might be the epigenetic post-translational inhibition/cleavage via the upregulated miRNAs identified. Some of them like miR‑17‑5p or miR‑222‑3p are already experimentally verified targets of PTEN while for others this work still has to be done [24]. All 32 miRNAs that we report to potentially target PTEN are upregulated and may therefore very well be accountable for the loss of the observed PTEN protein expression. In addition, all of them are significantly associated with a decreased RFS themselves in a Kaplan–Meier analysis.

### 3.5. VEGF

It has been known for a long time that VEGF plays a role in thyroid carcinomas [28] and first tyrosine kinase inhibitors also targeting VEGF have been successfully tested in patients [29].

VEGF has been linked to increased patient age [17] which we could confirm in the present work. We also found a loss of VEGF expression by immunohistochemistry to be associated with a decreased relapse free survival in univariate and multivariate analysis, including strong confounding factors like TC morphology. This finding is in contrast to previous reports claiming that an increased VEGF expression might predict increased local or distant recurrence [30,31]. One strength of the present study is the fact that we have included a large number of patients with an adverse clinical outcome. This enables us to overcome statistical limitations resulting in a too small number of patients with these rare adverse events. In addition, opposite results might also be partially due to different VEGF antibodies. Klein et al. used one which detected the isoform VEGF-A-206 which is not detected by the antibody used in the present study [30]—we detected the 165, 189, and 121 amino acid splice variants of VEGF.

A closer look at the VEGF gene itself is certainly worthwhile. The VEGF gene underlies differential splicing into several isoforms that differ from each other regarding the inclusion of the exons 6 and 7 into the transcript of the gene, which is responsible for the binding of the protein to extracellular matrix heparin. This results in the diffusible isoform VEGF-A121 at one end of the spectrum and in the strongly heparin binding isoform VEGF-A189 on the other [32]. Thus, it can be assumed that VEGF protein expression measured by immunohistochemistry corresponds to the tissue bound isoforms but does not provide any information concerning the presence and amount of the soluble isoforms. Since VEGF is secreted and high serum VEGF levels correlate with advanced tumor stages and lymph node metastases, it may thus well be possible that we observe a loss of VEGF expression on the immunohistochemical level in certain tumors because all VEGF has been secreted into the blood stream [33]. This would explain the high VEGF levels in serum and decreased VEGF expression by immunohistochemistry in patients who nevertheless might respond to VEGF inhibitors. Unfortunately, there is no study available correlating the immunohistochemical expression of VEGF with VEGF serum levels and in our patients, serum is not available in order to test this hypothesis.

### 3.6. PTEN, VEGF, TERT, TC and a Diagnostic Algorithm

The strong association of TERT promoter mutations and ACO has been well documented [7,34]. Here, we tested PTEN and VEGF in multivariate analyses together with TERT. Interestingly, all three were independent of TC morphology and were significantly able to predict an ACO. This gives us two new immunohistochemical tools to predict patient outcome, which is especially helpful since TERT promoter mutations occur only in a minority of 6–8% of cases and a reliable morphological diagnosis of TC is unfortunately still subjective and requires a lot of expertise. Although 10% of TC are already significantly associated with an ACO, different results are obtained by controlling for age, pT stage, gender, and TERT promoter mutation status. In this case, 20% TC is identified as the better overall cut-off to identify patients with a tumor relapse. This makes also sense from a morphological point-of-view, because a very low percentage of TC is inherently associated with false-positive cases. This is the moment, where VEGF and PTEN staining may come into place as they are not able to replace a thorough morphological workup, but their expression pattern can help to render a more accurate patient prognosis. Beyond morphology, implementing them into a diagnostic algorithm is mainly beneficial in order to predict tumor relapse in PTC with only a small percentage of TC which are in daily routine diagnostics, and indeed the difficult cases in the tumorboard decisions.

## 4. Material and Methods

### 4.1. Tissue Samples and Patient Characteristics

MiRNA profiling was performed on a patient cohort of 44 PTC (Table 7).

This cohort was part of a larger patient collective of 57 PTC with a relapse after initial thyroidectomy and radioiodine therapy (RAI) and an age-, stage- and gender-matched control group of 68 PTC without relapse. The details of this patient collective have been reported previously [7]. In short, the study collective was built in collaboration with the departments of nuclear medicine in the Canton Zurich (University Hospital Zürich, City Hospital Triemli, Cantonal Hospital Winterthur) where the patients‘ follow-up care was conducted and cases with a relapse could be identified. This enabled us to enrich the collective with tumors harboring an adverse outcome, overcoming statistical problems with low numbers of ACO patients. Tumors with a diameter of less than 1 cm were excluded from the analysis due to their indolent clinical course [35]. Surgical resection had been performed between 1990 and 2006, following RAI. The mean ± st.dev. follow-up-time for relapse free survival (RFS) was 51.78 ± 50.97 months.

The tissue of the study collective was taken from the archives of the corresponding Institutes of Pathology of the Canton Zurich; the tissue of the control collective was taken from the archives of the Institute of Pathology of the University hospital Zurich. After revision of the initial diagnosis by three board certified pathologists (MD, AS, AP) according to the 2017 WHO classification [25] and the current UICC TNM-classification (8th edition) [36], our study collective comprised a total of 125 PTCs including 57 cases with adverse clinical outcome (Table 6).

The study was conducted after approval of the responsible cantonal ethics committee (STV 28‑2006).

### 4.2. RNA Isolation and miRNA Expression Analysis

Tumor areas and non-neoplastic tissue areas with a high purity (>80%) were marked for microdissection on six blank consecutive slides (each 15 µm) under guidance of a stereomicroscope (Olympus SZ61, Hamburg, Germany). RNA was extracted with the RecoverAll kit (Ambion, Life Technologies, Carlsbad, CA, USA) and miRNA expression analysis was performed in tumorous (*n* = 44) and non-neoplastic (*n* = 8) thyroid FFPE tissue samples as previously described [11,12]. RNA quality and quantity was assessed with a spectrophotometer (NanoDrop 1000, Thermo Fisher Scientific, Wilmington, DE, USA), following pre-amplification and miRNA expression profiling using TaqMan RT-PCR microarray version v3.0 on the ABI 7900 platform which was designed to detect 754 human miRNA’s (Applied Biosystems Life Technologies, Carlsbad, CA, USA). RNU44 and U6 snRNA were used for normalization of RNA and non-human miRNA ath-miR-159a served as a negative control. miRNA expression levels were calculated using the 2-ΔΔct method using Dataassyst v3.1 software (Applied Biosystems). The maximum allowed Ct value for calculations was 37.

### 4.3. Immunohistochemical Analyses

A tissue micro array (TMA) was constructed as described previously [37] including a 0.6 mm core of tumor and normal tissue of histologically preselected regions of each case. A pathologist (MSD) blinded to the clinical data preselected tissue regions. Slides of 2 µm of the TMA were cut using a rotation microtome and immunohistochemical staining for PTEN, and VEGF-A protein expression was performed according to standard immunohistochemical techniques (Table S1). The VEGF antibody reacts with the 165, 189, and 121 amino acid splice variants of VEGF in humans. The markers were chosen by literature research [14,17]. The analysis of the immunohistochemical staining was conducted by a pathologist (MSD), who was blinded to the clinical data. The intensity of the staining (negative, weak, moderate, or strong) as well as the percentage of stained cells in the cytoplasm were recorded.

### 4.4. TERT Promoter Mutation Analysis

All tumors underwent TERT promoter mutation testing as described and previously reported [7].

### 4.5. Statistical Analysis

SPSS statistical software 24.0 (SPSS, Chicago, IL, USA) was used for assessment of the sample distribution (Kolmogorov–Smirnov test), the correlation of the clinicopathological data and the expression of the immunohistochemical markers with the Chi-Square test. For multivariate analysis (Cox regression) the factors age, pT-stage, gender, and TC morphology >10% were used as covariates. Survival analysis was calculated with Kaplan–Meier curves (log rank) and associations between miRNA expression and immunohistochemical data were calculated with the Kruskal–Wallis H test with Bonferroni correction. A *p*-value of <0.05 was considered statistically significant. MirDIP and miRTarBase were used for bioinformatic miRNA target prediction and identification of validated miRNA-protein-interactions [6,24].

## 5. Conclusions

Our data provides the most up-to-date comprehensive list of miRNAs linked to an adverse clinical outcome in PTC patients and lays the foundation for further informed basic research studies in this field. PTEN and VEGF are significant adverse prognostic factors in patients with PTC and a flow chart is proposed to identify patients with an adverse outcome. PTEN and VEGF are independent of other major prognostic factors including clinical features like age, tumor stage, and gender, molecular aberrations like TERT, and morphological factors like TCs. A variety of different miRNAs is highly likely to be responsible for this observation.

## Figures and Tables

**Table 1:** MiRNAs associated with decreased RFS (relapse free survival) and with TC (tall cell) morphology (bold).

MiRNA	*p*-Value (Mann *U* Test), Decreased RFS	*p*-Value (Mann *U* Test), Sign with TC-Morphology	Expression Level of miRNA Mean ± st. Error Mean
hsa-let-7a	0.001		1.68 ± 0.25
hsa-let-7c	0.001		1.01 ± 0.13
hsa-let-7f	0.013		1.34 ± 0.24
**hsa-let-7g**	**0.049**	**0.004**	**1.48 ± 0.31**
**hsa-miR-1**	**0.011**	**0.003**	**2.35 ± 0.63**
**hsa-miR-100-3p**	**0.001**	**0.004**	**3.77 ± 0.67**
**hsa-miR-106a-5p**	**0.017**	**0.002**	**1.58 ± 0.28**
hsa-miR-106b-3p	0.001		2.49 ± 0.46
hsa-miR-107	0.005		1.23 ± 0.26
**hsa-miR-1183**	**0.001**	**0.048**	**3.81 ± 0.76**
hsa-miR-1243	0.001		2.41 ± 0.40
hsa-miR-1253	0.007		7.76 ± 1.95
hsa-miR-1254	0.002		2.81 ± 0.95
hsa-miR-1255b	0.005		1.32 ± 0.20
**hsa-miR-1260**	**0.001**	**0.021**	**1.23 ± 0.19**
**hsa-miR-1262**	**0.001**	**0.05**	**20.07 ± 5.23**
**hsa-miR-126-5p**	**0.001**	**0.014**	**1.58 ± 0.34**
hsa-miR-1271	0.002		3.30 ± 0.87
hsa-miR-1274b	0.047		1.10 ± 0.15
hsa-miR-1276	0.001		1.87 ± 0.40
hsa-miR-128	0.001		1.40 ± 0.29
hsa-miR-1285	0.002		1.27 ± 0.20
**hsa-miR-1290**	**0.001**	**0.003**	**6.80 ± 1.88**
**hsa-miR-135b**	**0.001**	**0.002**	**4.04 ± 0.78**
**hsa-miR-136**	**0.001**	**0.0001**	**22.36 ± 6.26**
**hsa-miR-138**	**0.007**	**0.005**	**0.39 ± 0.07**
**hsa-miR-146b-5p**	**0.041**	**0.003**	**128.62 ± 23.62**
**hsa-miR-148a**	**0.028**	**0.029**	**0.53 ± 0.12**
hsa-miR-149	0.003		1.71 ± 0.37
hsa-miR-15b	0.001		1.68 ± 0.33
**hsa-miR-16**	**0.001**	**0.002**	**2.85 ± 0.60**
**hsa-miR-17**	**0.032**	**0.002**	**1.58 ± 0.28**
hsa-miR-181a-2-3p	0.013		5.97 ± 1.05
**hsa-miR-181c**	**0.001**	**0.0005**	**8.77 ± 3.18**
**hsa-miR-182**	**0.002**	**0.006**	**4.73 ± 1.56**
hsa-miR-1825	0.021		2.61 ± 0.45
hsa-miR-183-3p	0.006		5.13 ± 1.26
**hsa-miR-186**	**0.001**	**0.001**	**1.21 ± 0.23**
**hsa-miR-190**	**0.001**	**0.019**	**4.29 ± 2.88**
hsa-miR-190b	0.001		4.08 ± 0.81
**hsa-miR-191**	**0.041**	**0.007**	**1.44 ± 0.19**
**hsa-miR-195**	**0.001**	**0.001**	**1.08 ± 0.27**
**hsa-miR-196b**	**0.002**	**0.0004**	**4.56 ± 1.27**
**hsa-miR-197**	**0.004**	**0.001**	**2.05 ± 0.51**
hsa-miR-199a-5p	0.004		0.84 ± 0.15
hsa-miR-19b-1-5p	0.041		2.24 ± 0.45
**hsa-miR-200b**	**0.001**	**0.001**	**2.50 ± 0.47**
hsa-miR-202	0.001		16.08 ± 4.09
**hsa-miR-206**	**0.001**	**0.005**	**12.16 ± 2.33**
**hsa-miR-20a**	**0.046**	**0.003**	**1.58 ± 0.45**
hsa-miR-20a-3p	0.034		1.27 ± 0.34
**hsa-miR-21**	**0.002**	**0.001**	**7.50 ± 1.79**
hsa-miR-213	0.025		1.25 ± 0.19
hsa-miR-22	0.004		1.37 ± 0.31
hsa-miR-221	0.041		14.50 ± 2.27
**hsa-miR-222**	**0.005**	**0.004**	**22.37 ± 4.75**
**hsa-miR-223**	**0.046**	**0.0004**	**1.02 ± 0.18**
hsa-miR-22-5p	0.001		1.29 ± 0.23
hsa-miR-23b	0.006		1.54 ± 0.25
hsa-miR-24-2-5p	0.009		1.65 ± 0.23
**hsa-miR-26a**	**0.004**	**0.001**	**1.0 ± 0.17**
**hsa-miR-26b**	**0.001**	**0.0001**	**1.78 ± 0.48**
hsa-miR-26b-3p	0.028		2.05 ± 0.37
hsa-miR-28-3p	0.001		2.12 ± 0.46
hsa-miR-28-5p	0.001		0.81 ± 0.11
**hsa-miR-29a**	**0.03**	**0.006**	**2.33 ± 0.43**
**hsa-miR-29c**	**0.017**	**0.018**	**2.83 ± 1.26**
hsa-miR-301b	0.004		1.78 ± 0.49
hsa-miR-302d	0.022		1.58 ± 0.33
hsa-miR-30a-3p	0.003		0.98 ± 0.16
hsa-miR-30b	0.001		1.02 ± 0.14
hsa-miR-30c	0.032		0.92 ± 0.16
**hsa-miR-30d**	**0.039**	**0.009**	**1.25 ± 0.2**
hsa-miR-30e-3p	0.002		0.03 ± 0.17
**hsa-miR-32**	**0.001**	**0.003**	**16.04 ± 8.09**
hsa-miR-320	0.019		0.87 ± 0.09
**hsa-miR-337-5p**	**0.021**	**0.002**	**2.39 ± 0.69**
hsa-miR-339-3p	0.039		1.81 ± 0.31
**hsa-miR-340**	**0.001**	**0.001**	**2.24 ± 0.50**
hsa-miR-340-3p	0.001		1.92 ± 0.41
hsa-miR-34b	0.001		3.67 ± 0.75
**hsa-miR-361-5p**	**0.022**	**0.008**	**1.36 ± 0.25**
**hsa-miR-362-5p**	**0.012**	**0.009**	**1.19 ± 0.16**
**hsa-miR-372**	**0.007**	**0.043**	**11.12 ± 3.49**
hsa-miR-373	0.048		0.89 ± 0.23
**hsa-miR-374a**	**0.001**	**0.004**	**1.74 ± 0.47**
hsa-miR-374b	0.01		1.03 ± 0.15
**hsa-miR-376a**	**0.001**	**0.0003**	**3.30 ± 0.01**
hsa-miR-376a-5p	0.004		2.48 ± 0.55
**hsa-miR-376c**	**0.014**	**0.003**	**0.89 ± 0.21**
hsa-miR-380-5p	0.029		0.62 ± 0.19
**hsa-miR-411**	**0.028**	**0.003**	**0.89 ± 2.01**
hsa-miR-423-5p	0.019		2.08 ± 0.66
hsa-miR-425-3p	0.001		4.17 ± 1.16
**hsa-miR-429**	**0.001**	**0.002**	**4.39 ± 1.24**
**hsa-miR-454**	**0.001**	**0.003**	**1.85 ± 0.46**
**hsa-miR-483-5p**	**0.001**	**0.004**	**2.60 ± 0.46**
**hsa-miR-487a**	**0.003**	**0.05**	**10.96 ± 4.90**
hsa-miR-488	0.006		1.27 ± 0.26
**hsa-miR-489**	**0.001**	**0.017**	**2.99 ± 0.40**
**hsa-miR-493**	**0.022**	**0.022**	**1.58 ± 0.58**
**hsa-miR-494**	**0.001**	**0.003**	**3.31 ± 0.93**
hsa-miR-497	0.009		0.46 ± 0.09
hsa-miR-500	0.004		0.77 ± 0.10
hsa-miR-505-5p	0.022		1.96 ± 0.44
**hsa-miR-516-3p**	**0.001**	**0.023**	**4.98 ± 1.01**
hsa-miR-518b	0.001		14.93 ± 7.34
hsa-miR-518f	0.013		113.54 ± 73.78
**hsa-miR-520c-3p**	**0.001**	**0.035**	**4.94 ± 0.82**
hsa-miR-523	0.001		22.64 ± 5.71
hsa-miR-532-3p	0.004		0.83 ± 0.13
hsa-miR-532-5p	0.001		4.55 ± 1.81
**hsa-miR-539**	**0.002**	**0.01**	**4.35 ± 1.80**
**hsa-miR-545**	**0.001**	**0.004**	**3.15 ± 1.34**
hsa-miR-545-5p	0.007		15.62 ± 8.79
hsa-miR-548a-3p	0.001		17.74 ± 5.17
hsa-miR-548c-3p	0.001		21.88 ± 5.93
hsa-miR-571	0.038		2.94 ± 0.64
**hsa-miR-576-3p**	**0.001**	**0.002**	**2.47 ± 0.57**
**hsa-miR-577**	**0.001**	**0.027**	**1.34 ± 0.30**
hsa-miR-586	0.015		8.92 ± 2.37
**hsa-miR-590-3p**	**0.001**	**0.02**	**12.78 ± 6.23**
**hsa-miR-590-5p**	**0.001**	**0.004**	**4.46 ± 1.58**
hsa-miR-591	0.004		1.21 ± 0.28
hsa-miR-601	0.001		14.79 ± 2.70
hsa-miR-606	0.004		9.23 ± 2.69
**hsa-miR-625**	**0.002**	**0.0002**	**3.65 ± 0.89**
**hsa-miR-625-3p**	**0.001**	**0.024**	**6.99 ± 1.97**
**hsa-miR-627**	**0.001**	**0.0001**	**6.13 ± 1.54**
hsa-miR-628-5p	0.002		30.48 ± 14.89
hsa-miR-630	0.04		2.89 ± 0.85
**hsa-miR-636**	**0.001**	**0.001**	**1.10 ± 0.25**
**hsa-miR-645**	**0.008**	**0.011**	**1.13 ± 0.19**
hsa-miR-650	0.049		3.14 ± 1.12
**hsa-miR-655**	**0.025**	**0.004**	**4.95 ± 2.20**
**hsa-miR-661**	**0.001**	**0.015**	**1.99 ± 0.34**
hsa-miR-664	0.004		1.99 ± 0.36
hsa-miR-708	0.002		1.17 ± 0.23
hsa-miR-7-1	0.014		1.78 ± 0.32
hsa-miR-744-3p	0.001		1.55 ± 0.15
hsa-miR-758	0.001		4.81 ± 2.65
**hsa-miR-767-3p**	**0.011**	**0.029**	**10.70 ± 4.58**
**hsa-miR-885-5p**	**0.021**	**0.003**	**8.35 ± 5.17**
hsa-miR-892b	0.036		185.04 ± 156.31
**hsa-miR-92a**	**0.001**	**0.024**	**1.69 ± 0.38**
hsa-miR-92a-1-5p	0.008		0.77 ± 0.16
hsa-miR-93-3p	0.001		14.87 ± 7.51
**hsa-miR-95**	**0.025**	**0.015**	**1.78 ± 0.84**
hsa-miR-99b-3p	0.003		7.99 ± 1.78

**Table 2:** MiRNA predicted as target for vascular endothelial growth factor (VEGF) and with decreased RFS and PubMed Identifier (PMID) of miRNA–VEGF interaction validated in other papers.

miRNA Predicted as Target for VEGF and with Decreased RFS	*p* Value (Kaplan Meier, Log Rank)	Dichotomized at	VEGF as Target of Identified miRNA Validated in Other Experiment, PMID									
hsa-mir-1	0.052	1	27777493	28493075	27541266							
hsa-mir-106a-5p	0.028	1.4	18320040	23807165	18320040	17242205						
hsa-mir-1285-3p	0.011	1										
hsa-mir-17-5p	0.048	1	18320040									
hsa-mir-186-5p	0.000	1	27322147									
hsa-mir-195-5p	0.033	1	18320040	22473208	23592263	24398324	23446348	22012620	21572407	20371350	23468064	26823724
hsa-mir-200b-3p	0.000	2	21544626	28122882	25884496							
hsa-mir-202-3p	0.000	2										
hsa-mir-206	0.000	1	27541266									
hsa-mir-29c-3p	0.039	2	20371350	23592263	21572407	27000664						
hsa-mir-302d-3p	0.019	1	18320040									
hsa-mir-373-3p	0.003	0.5	18320040									
hsa-mir-374a-5p	0.003	0.5	23446348									
hsa-mir-429	0.001	2	26647818									
hsa-miR-494-3p	0.001	1										
hsa-mir-516a-3p	0.001	1										
hsa-mir-520c-3p	0.001	1										
hsa-mir-548a-3p	0.017	2										
hsa-mir-548c-3p	0.019	1										
hsa-mir-571	0.004	1										
hsa-mir-590-3p	0.001	1										
hsa-mir-645	0.013	1										

**Table 3:** MiRNA predicted as target for Phosphatase and tensin homolog (PTEN) and with decreased RFS and PMID of miRNA-PTEN interaction validated in other papers.

miRNA Predicted as Target for PTEN and with Decreased RFS	*p*-Value (Kaplan Meier, Log Rank)	Dichotomized at	PTEN as Target of Identified miRNA Validated in Other Experiment, PMID											
hsa-mir-106a-5p	0.028	1.4	28919047	24108762	26306906	26097565								
hsa-mir-1183	0.002	1												
hsa-mir-1255b-5p	0.009	1												
hsa-mir-1285-3p	0.011	1												
hsa-mir-1290	0.002	1												
hsa-mir-136-5p	0.000037	1												
hsa-mir-146b-5p	0.010	2												
hsa-mir-17-5p	0.048	1	20227518	20008935	21283765	20388916	23418359	24220575	24901013	24912422	24462867	22473208	23391506	23433743
hsa-mir-181c-5p	0.000055	2	25695913											
hsa-mir-186-5p	0.000	1												
hsa-mir-190b	0.009	1	26692329											
hsa-mir-200b-3p	0.000	2												
hsa-mir-222-3p	0.001	10	20618998	19962668	23028614	23974492	23994196	27644883						
hsa-mir-29c-3p	0.039	2	23143395											
hsa-mir-302d-3p	0.019	1												
hsa-mir-32-5p	0.001	2	23617834	22473208	24123284	23524257	28319142							
hsa-mir-337-5p	0.024	2												
hsa-miR-372-3p	0.035	3												
hsa-mir-373-3p	0.003	0.5												
hsa-mir-376a-3p	0.015	3												
hsa-mir-429	0.001	2	24866238											
hsa-miR-494-3p	0.001	1	20006626	22544933	25662849									
hsa-mir-520c-3p	0.001	1												
hsa-mir-539-5p	0.008	1.50												
hsa-mir-545-3p	0.000266	2												
hsa-mir-548a-3p	0.017	2												
hsa-mir-548c-3p	0.019	1												
hsa-mir-577	0.047	0.5												
hsa-mir-590-3p	0.001	1												
hsa-mir-628-5p	0.047	1												
hsa-miR-758-3p	0.026	0.3												
hsa-mir-767-3p	0.002	1												

**Table 4:** Correlation of VEGF and PTEN immunohistochemistry with miRNA expression and nr of miRNA Databases, predicting these interactions.

miRNA, Correlation with VEGF A	Kruskal–Wallis with Bonferroni Correction	*n* of Predicted miRNA Databases According to miRDIP
hsa-mir-142-3p	0.011	6
hsa-mir-142-5p	0.015	4
hsa-miR-155-3p	0.038	3
hsa-mir-193a-3p	0.041	3
hsa-mir-24-2-5p	0.038	3
hsa-miR-379-5p	0.013	4
hsa-miR-500a-5p	0.018	3
hsa-miR-630	0.047	4
hsa-mir-942-5p	0.037	12
**miRNA, Correlation with PTEN**		
hsa-miR-1-3p	0.023	8
hsa-miR-106a-5p	0.05	18
hsa-mir-17-5p	0.043	18
hsa-miR-190a-5p	0.025	8
hsa-miR-197-3p	0.035	6
hsa-miR-222-3p	0.004	10
hsa-miR-27b-3p	0.046	8
hsa-miR-372-3p	0.048	13
hsa-miR-381-3p	0.024	6
hsa-mir-508-3p	0.016	8
hsa-miR-642a-5p	0.035	11

**Table 5:** Correlation of relapse free survival and alteration of intensity in immunohistochemical staining (univariate analysis).

Immunohistochemical Marker		Control Group, *n* of Cases (Percentage of Cases)	Adverse Clinical Outcome Group, *n* of Cases (Percentage of Cases)	Probability Value
PTEN (cytoplasmic)	negative	0 (0%)	68 (54.8%)	0.026
	weak/moderate/strong	4 (3.2%)	52 (41.9%)	
VEGF A (cytoplasmic)	negative/weak	52 (42.3%)	16 (13%)	0.025
	moderate/strong	51 (41.5%)	4 (2.2%)	

**Table 6:** Clinicopathologic characteristics of the study collective with relapse after initial RAI (“adverse outcome”, ACO) and the control group (CG), TC (tall cell), chi-square test.

Demographics	Groups	Patients Total (*n* = 125)	Patients ACO (*n* = 57)	Patients CG (*n* = 68)	Probability Value
age	age <48	69 (55.2%)	32 (25.6%)	37 (29.6%)	*p* <0.859
	age ≥48	56 (44.8%)	25 (20.0%)	31 (24.8%)	
gender	male	34 (27.2%)	20 (16.0%)	14 (11.2%)	*p* <0.106
	female	91 (72.8%)	37 (29.6%)	54 (43.2%)	
pT-stage	pT 1–2	55 (44.0%)	18 (14.4%)	37 (29.6%)	*p* <0.012
	pT 3–4	70 (56.0%)	39 (31.2%)	31 (24.8%)	
TC	TC <10%	77 (61.6%)	21 (16.8%)	56 (44.8%)	*p* <0.001
	TC >10%	48 (38.4%)	36 (28.8%)	12 (9.6%)	

**Table 7:** Clinicopathologic characteristics of a subset of the study collective which underwent miRNA profiling; adverse outcome (ACO), control group (CG), TC (tall cell), chi-square test.

Demographics	Groups	Patients Total (*n*)	Patients ACO (*n* = 29)	Patients CG (*n* = 15)	Probability Value
age	age < 48	21 (47.7%)	15 (34.1%)	6 (13.6%)	*p* < 0.535
	age ≥ 48	23 (52.3%)	14 (31.8%)	9 (20.5%)	
gender	male	16 (36.4%)	11 (25%)	5 (11.4%)	*p* < 1
	female	28 (63.6%)	18 (40.9%)	10 (22.7%)	
pT-stage	pT 1–2	8 (18.2%)	5 (11.4%)	3 (6.8%)	*p* < 1
	pT 3–4	36 (81.8%)	24 (54.4%)	12 (27.3%)	
TC	TC <10%	20 (45.5%)	8 (18.2%)	12 (27.3%)	*p* < 0.001
	TC >10%	24 (54.5%)	21 (47.7%)	3 (6.8%)	

## Supplementary Materials

The following are available online at https://www.mdpi.com/2072-6694/11/6/885/s1, Table S1: Technical details of immunohistochemical stains.
