# Supplementary material for: MiRNAs Are Involved in Tall Cell Morphology in Papillary Thyroid Carcinoma
Source: Cancers (Basel). 2019 Jun 25;11(6):885. doi: 10.3390/cancers11060885 (PMC6628239; doi:10.3390/cancers11060885)
Supplement: Supplementary file 1 [file cancers-11-00885-s001.pdf]

Supplementary Materials

MiRNAs are Involved in Tall Cell Morphology in Papillary Thyroid Carcinoma

Laura A. Boos, Anja Schmitt, Holger Moch, Paul Komminoth, Cedric Simillion, Ilaria Marinoni, Yuri E. Nikiforov, Marina N. Nikiforova, Aurel Perren and Matthias S. Dettmer

Table S1. Technical details of immunohistochemical stains.

Antibody	Clone	Producer	Code	Technology	Dilution
PTEN	6H2.1	DAKO A/S	M3627	Bond 30/30 Refine	1: 200
VEGF A	polyclonal	NeoMarkers/Lab	RB-9031-P	UView DAB	1: 100

© 2019 by the authors. Licensee MDPI, Basel, Switzerland. This article is an open access article distributed under the terms and conditions of the Creative Commons Attribution (CC BY) license (http://creativecommons.org/licenses/by/4.0/).
